# Classification of Infected Necrotizing Pancreatitis for Surgery Within or Beyond 4 Weeks Using Machine Learning

**DOI:** 10.3389/fbioe.2020.00541

**Published:** 2020-06-04

**Authors:** Lan Lan, Qiang Guo, Zhigang Zhang, Weiling Zhao, Xiaoyan Yang, Huimin Lu, Zongguang Zhou, Xiaobo Zhou

**Affiliations:** ^1^West China Biomedical Big Data Center, West China Hospital, Sichuan University, Chengdu, China; ^2^Vascular Surgery, West China Hospital, Sichuan University, Chengdu, China; ^3^School of Information Management and Statistics, Hubei University of Economics, Wuhan, China; ^4^School of Biomedical Informatics, The University of Texas Health Science Center at Houston, Houston, TX, United States; ^5^Pancreatic Surgery, West China Hospital, Sichuan University, Chengdu, China; ^6^Institute of Digest Surgery, West China Hospital, Sichuan University, Chengdu, China

**Keywords:** classification, surgery, timing, machine learning, necrotizing pancreatitis

## Abstract

**Background:** The timing of surgery for necrotizing pancreatitis remains a matter of controversial debate, which has not been resolved by randomized controlled trial (RCT). This study aims to classify surgical timing within or beyond 4 weeks for patients with infected necrotizing pancreatitis by using machine learning methods.

**Methods:** This study analyzed 223 patients who underwent surgery for infected pancreatic necrosis at West China Hospital of Sichuan University. We used logistic regression, support vector machine, and random forest with/without the simulation of generative adversarial networks to classify the surgical intervention within or beyond 4 weeks in the patients with infected necrotizing pancreatitis.

**Results:** Our analyses showed that interleukin 6, infected necrosis, the onset of fever and C-reactive protein were important factors in determining the timing of surgical intervention (< 4 or ≥ 4 weeks) for the patients with infected necrotizing pancreatitis. The main factors associated with postoperative mortality in patients who underwent early surgery (< 4 weeks) included modified Marshall score on admission and preoperational modified Marshall score. Preoperational modified Marshall score, time of surgery, duration of organ failure and onset of renal failure were important predictive factors for the postoperative mortality of patients who underwent delayed surgery (≥ 4 weeks).

**Conclusions:** Machine learning models can be used to predict timing of surgical intervention effectively and key factors associated with surgical timing and postoperative survival are identified for infected necrotizing pancreatitis.

## Introduction

Necrotizing pancreatitis occurs in about 20% of patients suffering from acute pancreatitis (AP) (Banks, 1997). The current management guideline for necrotizing pancreatitis from IAP/APA (Working Group IAP/APA Acute Pancreatitis Guidelines, 2013) recommends delaying the timing of surgery until 4 or more weeks after initial necrotizing presentation to become walled-off shown in an addition file (Supplementary Figure 1). However, some patients with necrotizing pancreatitis will die before 4 weeks from the onset of AP. Therefore, how to identify those patients is an urgent problem to be solved. In addition to IAP/APA guideline, recommendations for surgical timing of necrotizing pancreatitis in the United States, United Kingdom, Italy, and Japan are also delayed as far as possible, without recommendations for individuals (Association et al., 2005; Tenner et al., 2013; Pezzilli et al., 2015; Yokoe et al., 2015).

Guo et al. concluded that the postoperative mortality of patients in 2 weeks with necrotizing pancreatitis was much higher than that after 2 weeks, and the prognosis of patients who did surgery before 4 weeks in necrotizing pancreatitis without persistent organ failure (POF) was same with that of patients who did surgery after 4 weeks in necrotizing pancreatitis without POF (Guo et al., 2014). A systematic review suggested that debridement should be done at least 12 days later for adult patients with necrotizing pancreatitis (Mowery et al., 2017). The first drainage time in step-up approach was 3.5–75.5 days from the onset of AP (Mowery et al., 2017). The timing of surgical intervention in necrotizing pancreatitis is controversial. A randomized controlled trial (RCT) which was established to optimize timings of surgery following PCD in patients with infected pancreatic necrosis was forced to stop early due to practical difficulties (Shenvi et al., 2016). The surgical timing problem has not been resolved by RCT.

What's more, infection and organ failure have long been used as key factors in determining whether or not to undergo surgery and are considered as the determinants of mortality for the patients with necrotizing pancreatitis. Surgical indications for the patients with necrotic pancreatitis are determined empirically among clinicians (Gomatos et al., 2015; Van Grinsven et al., 2016). A prospective study observed that POF in the first week was more likely to determine mortality than infection in patients with necrotizing pancreatitis (Guo et al., 2013). While a prospective cohort study from the Netherlands showed that there were no associations between infection, onset of organ failure, duration of organ failure and mortality in the patients with necrotizing pancreatitis (Schepers et al., 2018). These findings are inconsistent. Additionally, current studies cannot explain the relationship between the suggested surgical indications of necrotizing pancreatitis, mortality and surgical timing (Van Grinsven et al., 2016).

Nowadays, artificial intelligence (AI) is increasingly used in medicine (Nature Medicine, 2019). Therefore, we applied the machine learning and deep learning methods in AI to extract the clinical features from the patients with infected necrotizing pancreatitis who received early surgery in West China Hospital of Sichuan University and analyzed the associations between early surgical treatment, organ failure, infection and clinical predictors. We also identified the key factors associated with patients' mortality following early (<4 weeks) or late (≥4 weeks) surgery.

## Materials and Methods

### Patients and Treatment Protocol

A total of 223 patients (median age: 43 years old, male: 60.99%) were analyzed in this study. Those patients were hospitalized and operated due to infected necrotizing pancreatitis in West China Hospital of Sichuan University from January 2009 to June 2012. The AP was diagnosed according to the classification system of 2012 revision of the Atlanta edition, and pancreatic necrosis or peripancreatic necrosis was determined by contrast-enhanced computed tomography (CECT). Its treatment protocol was reported previously (Guo et al., 2013, 2014). The patients with severe clinical signs of persistent degeneration were operated before 4 weeks and the remaining patients were operated after 4 weeks from the onset of AP. This study was approved by the ethics review board of West China Hospital of Sichuan University, and the need for informed consent was waived owing to the retrospective nature of the study.

### Clinical Data Collection

The clinical data related above patients were collected, including infection, organ failure, operation time, postoperative mortality, postoperative complications, during hospitalization of those patients, etc. The collecting procedure and definitions of the indicators were described previously (Guo et al., 2013, 2014).

### Statistical Analysis

To classify surgical timing, there was nothing worthwhile to learn about a failed surgery. For example, if a patient died after surgery, we regarded this kind of case as a failed one. So, the successful surgery needed to be learned. We assumed that the best surgical timing was the actual time of a successful surgery. Based on the time from the onset of AP to surgical intervention, the patients were divided into the early (<4 weeks) and delayed (≥4 weeks) surgery groups. The baseline conditions of these patients were analyzed, including organ failure, infection, etc. *T*-test, and Chi-square test were used to evaluate the difference between the two groups. We then analyzed the factors that affect surgical timing and the factors associated with postoperative mortality by feature selection. Finally, we used multiple classifiers to classify the patients and compared the classifiers' performance. Variables with a *p* < 0.05 were considered to be statistically significant.

Three classifiers were used in this study, including logistic regression (LR), support vector machine (SVM) and random forest (RF) (Le, 2019; Le et al., 2019b). The LR is a commonly used statistic model in the healthcare industry and SVM is a popular machine learning approach. RF is a classifier that uses multiple trees to train and predict and has both features of high accuracy and balancing errors when analyzing unbalanced classification data sets. In order to find predictors of postoperative mortality at different surgical timings, in addition to feature selection and classification of surgical timing in survived patients after surgery, we performed feature selection and classification of postoperative death in the early and delayed surgery. Finally, we divided the patients into three groups based on the surgical time and mortality for classification analyses.

The survived patients after surgery (*n* = 186) were divided into the early group (*n* = 73) and the delayed group (*n* = 113), to predict whether surgical treatment should be performed early;

The patients received early surgery (*n* = 106) were divided into the death group (*n* = 33) and survival group (*n* = 73), to predict the death rate of patients after receiving an early surgery.

The patients with delayed surgery (*n* = 117) were divided into death group (*n* = 4) and survival group (*n* = 113), to predict the death rate of patients after delayed surgery.

**Figure 1:**
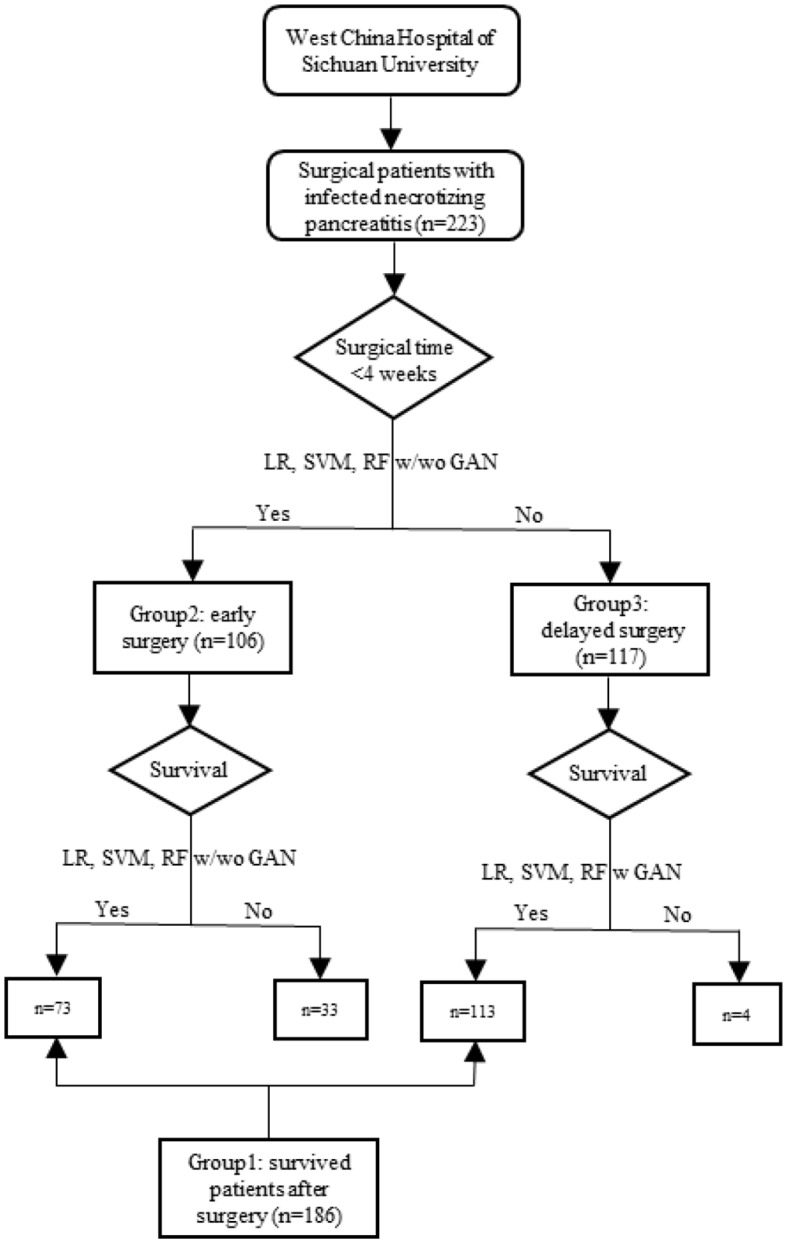
Flowchart of the study.

To solve the problem of positive and negative sample imbalance and small sample size, which will severely affect the performance of classifiers, we used generative adversarial networks (GAN) to generate simulated samples, which had the same distributions as the real samples (Creswell et al., 2017). GAN, a recently developed deep learning approach (Goodfellow et al., 2014), shows promising simulation performances in many fields (Deshpande, 2013; Santana and Hotz, 2016; Li et al., 2017; Pascual et al., 2017), such as image synthesis, language processing, etc. Douzas and Bacao (2017) used a conditional version (referring to each category) of GAN to approximate the true data distribution and generated data for the minority class of various imbalanced datasets. To improve the effectiveness of a classifier, Fiore et al. (2017) trained a GAN model to mimic the original minority class examples and then merged the synthetic examples with training data into an augmented training set. More importantly, by using variant of GAN, Baowaly et al. (2019) have proved that GAN can adequately learn the data distribution of real electronic health records and efficiently generate realistic synthetic electronic health records. GAN is a powerful generation model (Goodfellow et al., 2014; Douzas and Bacao, 2017; Fiore et al., 2017; Wang et al., 2017). Therefore, we applied GAN to electronic medical records to investigate the timing of surgical intervention for the patients with infected necrotizing pancreatitis. In this study, the data was randomly divided into training dataset and testing dataset according to the ratio of 4:1. The real training dataset were used to train the simulated samples to optimize GAN parameters. The simulated samples generated by the GAN generator were filtered by the GAN discriminator. The simulated samples after filtration were tested by LR, SVM, and RF (Figure 1).

We used several classification indicators to determine the classification performance of our models, including accuracy, precision, recall, F1-measure and area under curve (AUC) (Le et al., 2017, 2019a). Accuracy provides a percentage of correct classification. Precision is a measurement of how many positive classifications are actual positive observations. Recall, a proportion of all real positive observations that are correct, is a measure of how many actual positive observations are classified correctly. F1-measure, the harmonic mean of precision and recall, is an “average” of both precision and recall. AUC is the area under the ROC curve. The greater the value of the indicators, the better the model performance. We combined multiple evaluation indicators to evaluate the performance of the models. The simulation for GAN was calculated in Python software and others were conducted using R software.

## Results

### Characteristics of Survived Patients After Early or Delayed Surgery

We compared the major organ failure in the early and delayed surgery groups as shown in an additional file (Supplementary Table 1). In general, there were no differences in POF and the number of organ failure systems between the two groups. The proportion of renal failure in the early group was higher than that in the delayed group. Onsets of renal failure and multiple organ failure in the delayed group were earlier, but the duration of organ failure was shorter. In terms of the preoperative POF, more than half of patients with POF were recovered before surgery, and the proportion of POF in the early group was higher than that in the delayed group. As shown in another additional file (Supplementary Table 2), the median time and interquartile range of surgery for the early group were 21 and 6 days and the delayed group was 37 and 21 days, respectively. More patients received continuous renal replacement therapy (CRRT) in the early group than those in the delayed group. The delayed group had a higher proportion of infected necrosis. The onset of fever in the early group was earlier than that in the delayed group. The proportion of abnormal interleukin 6 (IL-6) level in the delayed group was higher than that in the early group. There was no difference in the modified Marshall score between the two groups, but preoperational modified Marshall score was higher in the early group. The proportions of intra-abdominal bleeding and re-intervention were higher in early group. Age, length of hospital stay and gender composition ratio were similar between the two groups. There were no differences between the two groups in blood culture, sputum, white blood cell (WBC), C-reactive protein (CRP), procalcitonin (PCT), enterocutaneous fistula, and new-onset organ failure.

### Predictors of Surgical Timing and Postoperative Mortality and Classification Performance

In order to classify the surgical timing (<4 or ≥4 weeks), the patients were divided into three groups as shown in the Methods based on the time of surgery and postoperative mortality, including the patients surviving after surgery (group 1), the patients received earlier surgery (group 2) and the patients underwent delayed surgery (group 3). LR, SVM, RF w/wo GAN were adopted to predict surgical timing from the three groups of patients, respectively, where LR was used as a statistical model and SVM and RF were used as machine learning models. The first group of patients was used to assess the predictors of surgical timing. The second group of patients was used for evaluating the predictors of mortality from early surgery and the third group of patients for the predictors of mortality from delayed. We used the stepwise selection procedures for the selection of independent variables (predictors) in LR. The Boruta function in R was applied to select important features in SVM, where the value of *meanImp* indicates the importance of a predictor. RF itself comes with a feature selection function, where the value of *MeanDecreaseGini* represents the importance of a feature. The larger the value, the more important it is.

The analysis results for group 1 indicated that IL-6, infected necrosis, the onset of fever and CRP were the important factors for surgical timing of patients with necrotizing pancreatitis (Table 1). The results of the following models are derived from the testing dataset. We also assessed the classification accuracy of the three models. The classified accuracy of RF (0.80) was higher than SVM (0.78) and LR (0.71). With the simulation by the GAN, the classification accuracies for all of the three models were improved. GAN-RF (accuracy: 0.89) had a better performance than GAN-SVM (0.84) and GAN-LR (0.83). The recall rates also reached 1 (Table 2).

**Table 1:** Top important features for survived patients after early surgery (<4 weeks) compared with survived patients after delayed surgery (≥4 weeks).

**LR**		**SVM**		**RF**	
**Significant variable**	** 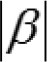 **	**Confirmed variable**	***meanImp***	**Variable**	***MeanDecreaseGini***
Pulmonary failure	21.89	Onset of fever	21.40	Onset of fever	14.25
POF	19.77	Infected necrosis	13.37	Age	9.18
Renal failure	5.11	IL-6	10.82	Infected necrosis	5.10
IL-6	2.71	Modified Marshall score pre-operation	9.58	Modified Marshall score on admission	4.35
PCT	1.36	Modified Marshall score on admission	7.99	CRP	3.54
Duration of organ failure	1.20	Sputum	7.63	IL-6	3.40
Infected necrosis	1.11	CRRT	6.79	Modified Marshall score pre-operation	3.31
WBC	0.78	CRP	6.12	Duration of organ failure	2.66
Onset of fever	0.68	Onset of renal failure	5.49	WBC	1.63
CRP	0.31	Renal failure	5.42	Sputum	1.62

*LR, logistic regression; SVM, support vector machine; RF, random forest; POF, persistent organ failure; IL-6, interleukin 6; PCT, procalcitonin; WBC, white blood cell; CRP, C-reactive protein; CRRT, continuous renal replacement therapy*.

**Table 2:** Classification performance for survived patients after early (<4 weeks) or delayed surgery (≥4 weeks).

**Model**	**Accuracy**	**Precision**	**Recall**	**F1-Measure**	**AUC**
LR	0.71	0.70	0.53	0.58	0.71
SVM	0.78	0.77	0.63	0.67	0.75
RF	0.80	0.75	0.70	0.71	0.78
GAN-LR	0.83	0.62.	1.00	0.76	0.90
GAN-SVM	0.84	0.72	1.00	0.84	0.86
GAN-RF	0.89	0.80	1.00	0.88	0.90

*LR, logistic regression; SVM, support vector machine; RF, random forest; GAN, generative adversarial networks*.

We assessed the key factors affecting patient mortality in the group 2 patients using LR, SVM, and FR models, respectively. As shown in the Table 3, top-ranked factors associated with patient mortality include the modified Marshall score on admission and preoperational modified Marshall score. By combining with GAN, the classification accuracies of the three models for mortality in early surgery patients were largely improved. GAN-RF (0.99) and GAN-SVM (0.99) had a better performance in evaluating the key factors than GAN-LR (0.90) (Table 4).

**Table 3:** Top important features for mortality after early surgery (<4 weeks).

**LR**		**SVM**		**RF**	
**Significant variable**	** 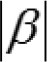 **	**Confirmed variable**	***meanImp***	**Variable**	***MeanDecreaseGini***
CRRT	744.77	Renal failure	10.32	Modified Marshall score on admission	5.76
Intra-abdominal bleeding	424.34	Onset of renal failure	10.19	Renal failure	4.87
Blood culture	373.11	Onset of fever	9.87	Onset of multiple organ failure	4.70
New-onset organ failure	297.63	Re-intervention	9.38	Onset of renal failure	3.97
Modified Marshall score on admission	147.89	Modified Marshall score on admission	9.02	Number of organ failure systems	3.23
POF pre-operation	134.08	Onset of multiple organ failure	7.54	Modified Marshall score pre-operation	2.74
Modified Marshall score pre-operation	97.74	Number of organ failure systems	7.51	Onset of fever	1.78
WBC	75.50	Multiple organ failure	7.45	Re-intervention	1.71
PCT	72.39	POF pre-operation	6.68	Duration of organ failure	1.62
Age	0.35	Modified Marshall score pre-operation	6.48	Age	1.62

*LR, logistic regression; SVM, support vector machine; RF, random forest; POF, persistent organ failure; PCT, procalcitonin; WBC, white blood cell; CRRT, continuous renal replacement therapy*.

**Table 4:** Classification performance for mortality after early surgery (<4 weeks).

**Model**	**Accuracy**	**Precision**	**Recall**	**F1-Measure**	**AUC**
LR	0.90	0.82	0.88	0.83	0.94
SVM	0.94	0.94	0.90	0.91	0.93
RF	0.94	0.85	1.00	0.90	0.96
GAN-LR	0.90	0.89	0.92	0.90	0.97
GAN-SVM	0.99	0.99	0.99	0.99	0.99
GAN-RF	0.99	0.99	0.99	0.99	0.99

*LR, logistic regression; SVM, support vector machine; RF, random forest; GAN, generative adversarial networks*.

As shown in the Table 5, the modified Marshall score pre-operation was predicted by all three models as an important factor for the mortality of patients who underwent delayed surgery. The time of surgery, duration of organ failure and onset of renal failure were top 5-ranked features predicted by SVM and RF models (Table 6). Due to the unbalanced positive and negative samples, we simulated this group of samples using GAN first and then did classification analysis for the postoperative mortality using three classifiers. The classification accuracies of GAN-LR, GAN-SVM, and GAN-RF were 0.97, 0.99, and 0.99, respectivel (Table 6).

**Table 5:** Top important features for mortality after delayed surgery (≥4 weeks).

**LR**		**SVM**		**RF**	
**Significant variable**	** 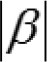 **	**Confirmed variable**	***meanImp***	**Variable**	***MeanDecreaseGini***
Circulatory failure	60.16	Time of surgery	12.13	Time of surgery	167.62
Modified Marshall score pre-operation	14.17	Duration of organ failure	9.52	Duration of organ failure	122.11
		Modified Marshall score pre-operation	8.64	Onset of renal failure	111.15
		Onset of renal failure	8.60	Onset of fever	108.94
		Male	8.35	Modified Marshall score pre-operation	66.56
		Onset of multiple organ failure	7.34	Onset of multiple organ failure	52.52
		Onset of fever	7.30	Onset of single organ failure	45.50
		Modified Marshall score on admission	7.19	Onset of POF	45.18
		Number of organ failure systems	7.17	Male	43.82
		Blood culture	6.99	POF pre-operation	32.31

*LR, logistic regression; SVM, support vector machine; RF, random forest; POF, persistent organ failure*.

**Table 6:** Classification performance for mortality after delayed surgery (≥4 weeks).

**Model**	**Accuracy**	**Precision**	**Recall**	**F1-Measure**	**AUC**
GAN-LR	0.97	0.69	0.70	0.63	0.92
GAN-SVM	0.99	0.80	1.00	0.88	0.99
GAN-RF	0.99	0.94	0.99	0.96	0.99

*LR, logistic regression; SVM, support vector machine; RF, random forest; GAN, generative adversarial networks*.

## Discussion

This study has two main highlights. (1) We compared the performance of machine learning models with a common statistic model (LR) and the performance of machine learning models were better. (2) We identified the key factors associated with surgical timing (<4 or ≥4 weeks) and postoperative survival for infected necrotizing pancreatitis and predicted the surgical timing by applying machine learning models.

An international survey shows that 55% of pancreatic specialists would wait for the effect of antibodies and postpone surgical management for the patients with infected pancreatic necrosis, whereas 45% of specialists would take an immediately action of surgical treatment after diagnosis (Abdelhafez et al., 2015). The time of operation varies greatly. Therefore, it is necessary to demonstrate if the patient with necrotizing pancreatitis needs early or delayed surgery individually. Previous studies using organ failure and infection as predictors of death obtained controversial results (Guo et al., 2013, 2014; Schepers et al., 2018). In our study, we assessed the impact of multiple clinical factors and comprehensive scores on surgical timing and postoperative mortality for the patients received the early or delayed surgery.

Early studies showed that the mortality of patients who received surgery within 2 weeks was much higher than that of surgery after 2 weeks (Besselink et al., 2007; Guo et al., 2013, 2014; Schepers et al., 2018), suggesting that early surgery should be conducted between 2 and 4 weeks. According to our analysis with multiple classifiers, IL-6, infected necrosis, onset of fever and CRP are important factors associated with the timing of surgery, which is consistent with the surgical indications used in clinic. The modified Marshall score is one of common factors used to assess patient mortality. Our analysis indicated that the mortality of the early surgery group was associated with the preoperative modified Marshall score and the modified Marshall score assessed at admission, suggesting that the modified Marshall score should be monitored in a real time for prediction. According to the Revised Atlanta Classification, organ failure is determined by modified Marshall score. The preoperative modified Marshall score was associated with the mortality after delayed surgery. Only two meaningful variables were obtained through stepwise regression of LR, including the preoperative modified Marshall score and circulatory failure. The preoperative modified Marshall score, the time of surgery, duration of organ failure and onset of renal failure were among of the top five important features selected by SVM and RF. A recent multicenter prospective cohort study reported that POF and multiple organ failure were the major determinants of AP severity, and the presence of infection was not associated with higher mortality (Sternby et al., 2017), consistent with our findings.

According to our knowledge, this is the first time to apply SVM and RF to predict the timing of surgery and postoperative mortality of patients with infected necrotizing pancreatitis. The classification performance of RF and SVM was better than LR. Especially when GAN was applied in the simulation, the accuracies were obviously improved. It is most likely because GAN can generate simulation samples with the same distribution as the actual samples, enhancing the sample size. In term of model classification performance, the classification accuracies of three models were high. Therefore, based on the patient's routine laboratory test and organ failure status, we can apply the classifiers to predict whether the patient should undergo early or delayed surgery individually to reduce patient mortality. Our classification results provide good references for clinicians to make personized surgical plans for patients with infected necrotizing pancreatitis.

However, there are some limitations of this study. Since the categorical variables cannot be applied to the traditional GAN, we changed the categorical variables into continuous variables and then put them into the GAN model. Although we have reached a conclusion consistent with Baowaly et al. by using our proposed GAN, we need to further verify with more samples.

In summary, we (1) applied a better machine learning model compared with a statistic model to predict the surgical timing (<4 or ≥4 weeks) in patients with infected necrotizing pancreatitis; (2) identified the key factors associated with surgical timing and postoperative survival for infected necrotizing pancreatitis and predicted the surgical timing by applying machine learning models; and (3) provided good references for clinicians in developing personalized surgical plans for patients with infected necrotizing pancreatitis.

## Data Availability Statement

The datasets generated during and/or analyzed during the current study are available from the corresponding author on reasonable request.

## Ethics Statement

This study was approved by the ethics review board of West China Hospital of Sichuan University, and the need for informed consent was waived owing to the retrospective nature of the study.

## Author Contributions

Data collection: QG. Data analysis: LL and ZZha. Data interpretation: XY and HL. Writing of the manuscript: LL. Research conception: XZ and ZZho. Critical revision of the manuscript: WZ.

## Conflict of Interest

The authors declare that the research was conducted in the absence of any commercial or financial relationships that could be construed as a potential conflict of interest.
