# Supplementary material for: Classification of Infected Necrotizing Pancreatitis for Surgery Within or Beyond 4 Weeks Using Machine Learning
Source: Front Bioeng Biotechnol. 2020 Jun 4;8:541. doi: 10.3389/fbioe.2020.00541 (PMC7287166; doi:10.3389/fbioe.2020.00541)
Supplement: Supplementary file 1 [file Data_Sheet_1.pdf]

Supplementary Material

Supplementary Figure 1. The diagnosis and intervention of infected necrotizing pancreatitis.

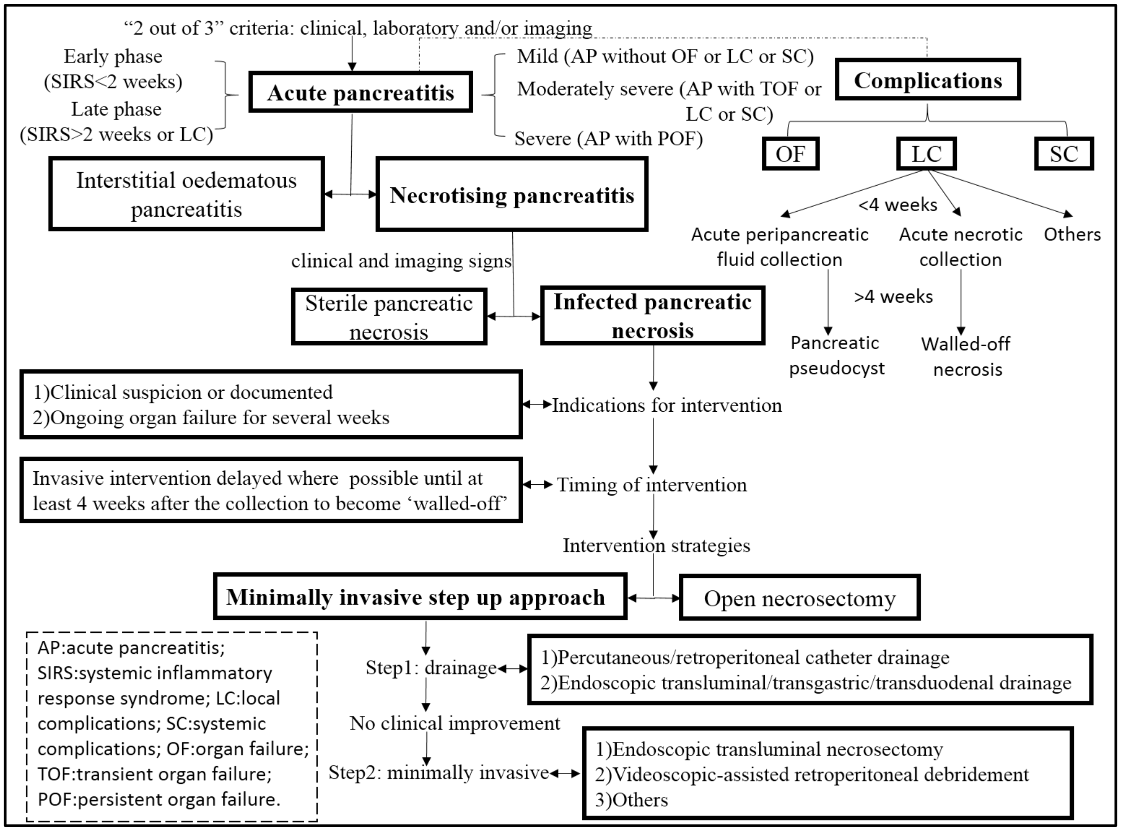

Supplementary Table 1. Preoperational organ failure analyses for survived patients after early (<4 weeks) or delayed surgery (≥ 4 weeks).

POF	All[a] (*N*=186)	<4 weeks[b] (*N*=73)	≥4 weeks[b] (*N*=113)	p
POF, N(%)	79(42.47)	34(46.58)	45(39.82)	0.363
Number of organ failure systems				
One organ system, N(%)	55(69.62)	20(58.82)	35(77.78)	
Two organ systems, N(%)	18(22.78)	9(26.47)	9(20.00)	0.099
Three organ systems, N(%)	6(7.59)	5(14.71)	1(2.22)	
Pulmonary failure, N(%)	73(39.25)	34(46.58)	39(34.51)	0.100
Renal failure, N(%)	20(10.75)	12(16.44)	8(7.08)	0.044
Circulatory failure, N(%)	16(8.60)	7(9.59)	9(7.96)	0.700
Onset of POF				
First week(>48 hours), N(%)	72(92.31)	34(100)	38(86.36)	
Second week, N(%)	3(3.85)	0(0)	3(6.82)	0.102
Third or fourth week, N(%)	0(0)	0(0)	0(0)	
Fifth week or later, N(%)	3(3.85)	0(0)	3(6.82)	
Onset of pulmonary failure, M(IQR)	3(3)	3(1)	3(3)	0.101
Onset of renal failure, M(IQR)	2(1)	3(1)	2(1)	0.044
Onset of circulatory failure, M(IQR)	3(2)	3(2)	3(1)	0.700

Duration of organ failure, M(IQR)	14(16)	14.5(10)	9(22)	
48 hours to 1 week, N(%)	32(41.56)	11(32.35)	21(48.84)	
1–2 weeks, N(%)	8(10.39)	6(17.65)	2(4.65)	0.028
2–3 weeks, N(%)	32(41.56)	17(50.00)	15(34.88)	
>3 weeks, N(%)	5(6.49)	0(0)	5(11.63)	
Single organ failure, N(%)	55(29.57)	20(27.40)	35(30.97)	0.102
Onset of single organ failure				
First week(>48 hours), N(%)	72(92.31)	34(100)	38(86.36)	
Second week, N(%)	3(3.85)	0(0)	3(6.82)	0.774
Third or fourth week, N(%)	0(0)	0(0)	0(0)	
Fifth week or later, N(%)	3(3.85)	0(0)	3(6.82)	
Multiple organ failure, N(%)	24(12.90)	14(19.18)	10(8.85)	0.056
Onset of multiple organ failure				
First week(>48 hours), N(%)	22(91.67)	12(85.71)	10(100)	
Second week, N(%)	2(8.33)	2(14.29)	0(0)	0.021
Third or fourth week, N(%)	0(0)	0(0)	0(0)	
Fifth week or later, N(%)	0(0)	0(0)	0(0)	
POF pre-operation, N(%)	30(16.13)	17(23.29)	13(11.50)	0.033

POF, persistent organ failure; M(IQR), median and inter-quartile range for quantitative data; N(%), number and percentage for categorical variables; a indicates the survival after operation; b indicates the time from the onset of acute pancreatitis to surgical intervention.

Supplementary Table 2. Infection and other baseline characteristics for survived patients after early (<4 weeks) or delayed surgery (≥ 4 weeks).

Characteristic	All[a] (*N*=186)	<4 weeks[b] (*N*=73)	≥4 weeks[b] (*N*=113)	p
Age(years), M(IQR)	43(17)	41(12)	45(17)	0.166
Male, N(%)	115(61.83)	45(61.64)	70(61.95)	0.967
Length of stay(days), M(IQR)	25(32)	24(30)	26(34)	0.931
Time (days) from onset to surgery, M(IQR)	32(20)	21(6)	37(21)	<0.001
CRRT, N(%)	17(9.14)	12(16.44)	5(4.42)	0.006
Infected necrosis, N(%)	99(53.23)	26(38.36)	71(62.83)	0.001
Onset of fever(days), M(IQR)	7(15)	7(11)	9(30)	<0.001
Blood culture, N(%)	16(8.60)	7(9.59)	9(7.96)	0.700
Sputum, N(%)	30(16.13)	10(13.70)	20(17.70)	0.469
WBC, N(%)	71(38.17)	30(41.10)	41(36.28)	0.509
CRP				
Normal, N(%)	78(41.94)	28(38.36)	50(44.25)	
Mild, N(%)	4(2.15)	0(0)	4(3.54)	0.206
Moderate, N(%)	13(6.99)	4(5.48)	9(7.96)	
Severe, N(%)	91(48.92)	41(56.16)	50(44.25)	
PCT, N(%)	55(29.57)	24(32.88)	31(27.43)	0.427
IL-6, N(%)	44(23.66)	11(15.07)	33(29.20)	0.027

Modified Marshall score on admission, M(IQR)	1(3)	1(3)	1(2)	0.122
Modified Marshall score pre-operation, M(IQR)	0(1)	0(1)	0(0)	0.009
Postoperative complications				
Intra-abdominal bleeding, N(%)	23(12.37)	16(21.92)	7(6.19)	0.002
Enterocutaneous fistula, N(%)	20(10.75)	9(12.33)	11(9.73)	0.577
New-onset organ failure, N(%)	14(7.53)	8(10.96)	6(5.31)	0.154
Re-intervention, N(%)	19(10.22)	17(23.29)	2(1.77)	<0.001

M(IQR), median and inter-quartile range for quantitative data; N(%), number and percentage for categorical variables; CRRT, continuous renal replacement therapy; WBC, white blood cell; CRP, C-reactive protein; PCT, procalcitonin; IL-6, interleukin 6; a indicates the survival after operation; b indicates the time from the onset of acute pancreatitis to surgical intervention.
